# Signaling Mechanisms by Arabidopsis Cryptochromes

**DOI:** 10.3389/fpls.2022.844714

**Published:** 2022-02-28

**Authors:** Jathish Ponnu, Ute Hoecker

**Affiliations:** Institute for Plant Sciences and Cluster of Excellence on Plant Sciences (CEPLAS), Biocenter, University of Cologne, Cologne, Germany

**Keywords:** cryptochromes, CRY1, CRY2, Arabidopsis, blue light, signal transduction, photomorphogenesis

## Abstract

Cryptochromes (CRYs) are blue light photoreceptors that regulate growth, development, and metabolism in plants. In *Arabidopsis thaliana* (Arabidopsis), CRY1 and CRY2 possess partially redundant and overlapping functions. Upon exposure to blue light, the monomeric inactive CRYs undergo phosphorylation and oligomerization, which are crucial to CRY function. Both the N- and C-terminal domains of CRYs participate in light-induced interaction with multiple signaling proteins. These include the COP1/SPA E3 ubiquitin ligase, several transcription factors, hormone signaling intermediates and proteins involved in chromatin-remodeling and RNA N6 adenosine methylation. In this review, we discuss the mechanisms of Arabidopsis CRY signaling in photomorphogenesis and the recent breakthroughs in Arabidopsis CRY research.

## Introduction

As autotrophic organisms, plants use light as their source of energy through photosynthesis. Light is therefore of uttermost importance to plants and other photosynthesizing organisms. Plants evolved intricate mechanisms to adjust growth, development, and metabolism to the ambient light environment, which varies considerably throughout the days and seasons. Such adjustment optimizes growth under low-light conditions—to increase photosynthesis and competitiveness—as well as under excess light conditions—to prevent oxidative damage caused by high light or UV radiation. Because plants are sessile and, therefore, cannot move to a “better” place when local light conditions are suboptimal, their survival and fitness heavily depend on adequate adaptation to the varying ambient light conditions at their site. Plants, therefore, exhibit enormous plasticity in their growth and development, showing very different phenotypes under different environmental conditions. Hence, genetic developmental programs are not static but are very strongly regulated by light (Kami et al., 2010; Huber et al., 2020).

To sense the ambient light, plants evolved several classes of photoreceptors. These photoreceptors exist in almost every cell of the plant, allowing these cells to individually sense and respond to light. In addition, photoreceptors induce systemic signals that allow communication across cells and tissues to coordinate the growth and development of the entire plant. Photoreceptors enable plants to sense multiple pieces of information beyond the intensity of light, that is color, direction, and daylength (photoperiod). To do so, specialized photoreceptors evolved. These include several classes of blue light receptors: the cryptochromes (CRYs; covered in this review), the phototropins responsible for directional growth towards the light (phototropism), chloroplast movement within the cell and opening of stomata, which are the air pores in plant leaves, and the ZEITLUPE family of photoreceptors (Christie et al., 2015). Red and far-red light are sensed by phytochromes which enable plants to specifically sense and respond to competing, neighboring plants (Cheng et al., 2021). UV-B light is perceived by the UV-B receptor UVR8, having a major role in photomorphogenesis and providing photoprotection from damaging UV light (Podolec et al., 2021).

While light responses have been studied in multiple plant species including crops, most of what we know about photoreceptor functions and—in particular—signaling events is derived from research in the model species *Arabidopsis thaliana* (Arabidopsis). It is a small, rapid-cycling weed with superior availability of molecular tools, and low-cost access to mutants in almost every gene through the Arabidopsis stock centers. Besides, researchers worldwide can make use of several freely accessible databases for *in silico* DNA, RNA, and protein analyses in Arabidopsis (Garcia-Hernandez and Reiser, 2006; Berardini et al., 2015; The 1001 Genomes Consortium, 2016; Zhang et al., 2020). This review will therefore focus on our current knowledge of Arabidopsis CRYs, with special attention to very recent research progress. For a more comprehensive review of CRY functions, the reader is referred to a previous excellent review (Wang and Lin, 2020a). Our review complements other reviews in this Frontiers collection, such as light-sensing through CRY chromophores (Goett-Zink and Kottke, 2021).

## Cryptochromes Regulate Arabidopsis Growth, Development, Metabolism, and Stress Tolerance

**Figure 1:**
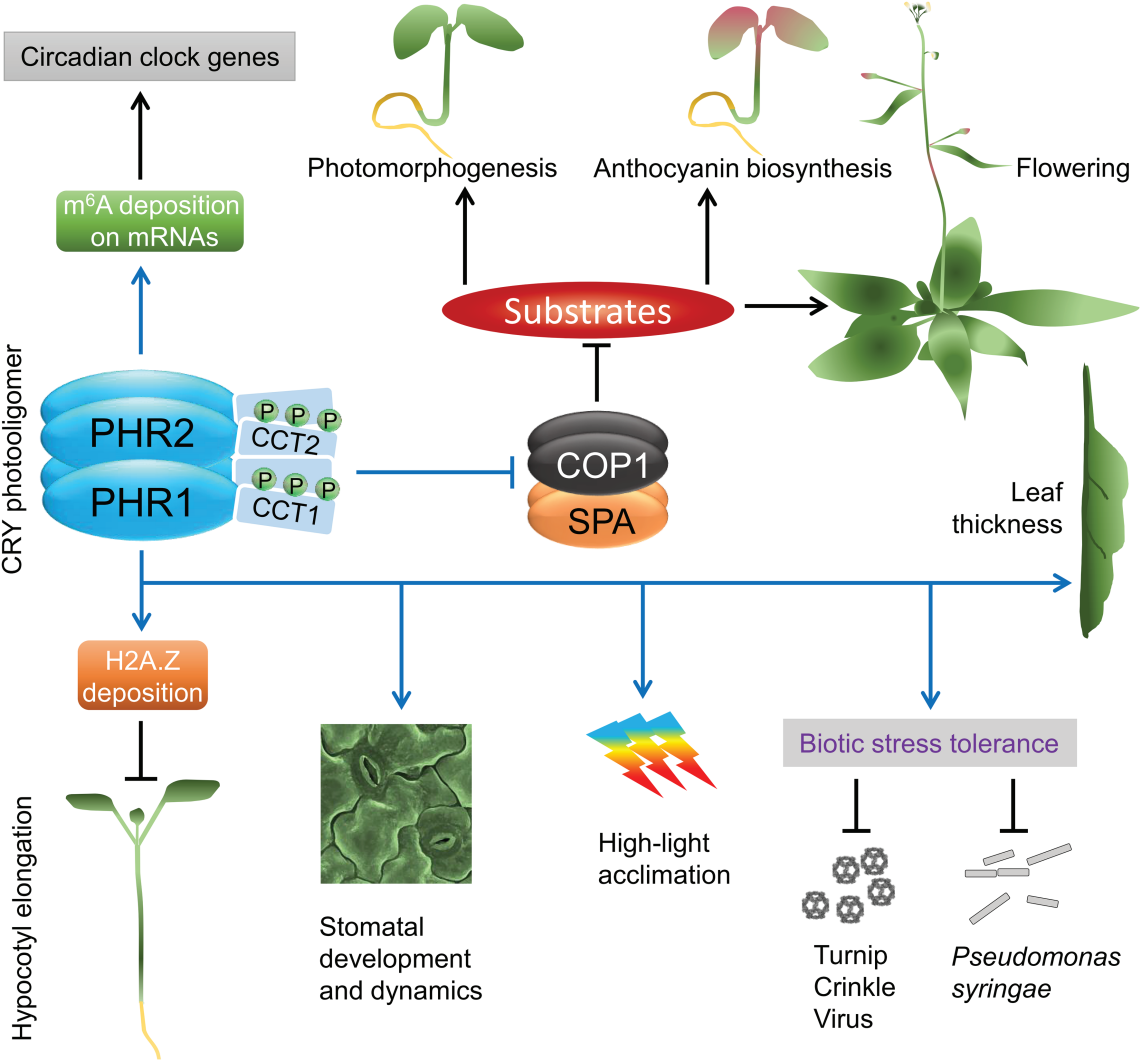
An overview of cryptochrome signaling in Arabidopsis. Blue lines and arrows represent the direct involvement of CRYs. Abbreviations: PHR, photolyase homology region; CCT, CRY carboxy terminus; COP1, CONSTITUTIVE PHOTOMORPHOGENIC 1; SPA, SUPPRESSOR OF PHYA-105 1; m^6^A, methylation of N6 adenosine; H2A.Z, a variant of histone H2A.

In response to blue light, CRYs regulate almost every stage in the plant life cycle, from seedling establishment to the induction of reproductive growth (Figure 1). A most pronounced phenotype is observed in germinating seedlings. This conspicuous phenotype was therefore widely used in mutant screens that allowed the isolation of many genes important for the light response, including the CRY photoreceptors and CRY signaling components.

A germinating seedling is supported by storage reserves in the seed only for a few days after which it needs to start photosynthesis to survive and sustain further growth. This process is called deetiolation and includes the opening of closed embryonic leaves (cotyledons), the differentiation of initially immature chloroplasts into organelles fully capable of photosynthesis, the biosynthesis of chlorophyll pigment as well as the shortening of the embryonic stem (hypocotyl). Deetiolation is induced by blue light through CRYs and by red light through phytochrome photoreceptors. When a seedling grows in continuous darkness, that is, when covered by soil or litter, the default pathway is etiolation: the seedling uses all reserves stored in the seed for elongation to grow through the cover and reach the sunlight. During this period, the hypocotyl elongates to more than 10 times the length of a light-grown seedling—exclusively *via* enhanced cell elongation (Gendreau et al., 1997). Elongation occurs at the expense of most other processes; cotyledons remain closed and chloroplasts do not differentiate nor green. Moreover, a so-called apical hook forms to protect the shoot apical meristem and the cotyledons from damage while the seedling pushes through the soil (Kami et al., 2010; Mazzella et al., 2014).

Deetiolation in the light is achieved by a dramatic change in gene expression involving 10–20% of all genes (Peschke and Kretsch, 2011). These genes are ultimately CRY- and phytochrome-regulated genes and include genes involved in photosynthesis, chloroplast formation, and cell elongation (Griffin et al., 2020). Most of these genes are also under the control of the circadian clock. Recent reports suggest that CRYs play an important role in the entrainment of the circadian clock to the day/night cycle and the burst of gene expression during early dawn (Balcerowicz et al., 2021; Lopez et al., 2021). While phototropins are the blue light receptors for the directed growth of plants toward the light, CRY1 has recently been shown to modulate the phototropic curvature of seedlings in blue light (Boccaccini et al., 2020).

Once a seedling is established, it continues to grow and develop *via* the division of meristematic cells and the production of primordia. Cell division in the shoot apical meristem and leaf primordia requires inputs from light (*via* CRY or phytochrome photoreceptors in blue and red light, respectively) and sugar signals. Hence, seedlings placed in darkness fail to develop any further, not even when supplied with sucrose as an external energy source (Lopez-Juez et al., 2008; Yoshida et al., 2011; Pfeiffer et al., 2016). Leaf primordia subsequently develop into leaves whose thickness is influenced by the light intensity. There is good evidence that the increased thickness of leaves induced by higher light intensities is dependent on CRYs in co-action with phototropins (Weston et al., 2000; Hoshino et al., 2019). During the vegetative phase, Arabidopsis develops a rosette whose leaves are arranged perpendicular to the incoming light, thereby allowing maximal light absorption for photosynthesis. However, when plants are grown under low blue light intensities which limit CRY activity, they undergo a shade avoidance response exhibiting increased elongation of the petioles and a lifting of the leaves (hyponasty). This change in phenotype increases light exposure under plant–plant competition in dense canopies (Keller et al., 2011; De Wit et al., 2016; Pedmale et al., 2016). CRYs are also involved in the protection of plants from excess light by inducing the biosynthesis of substances that protect the photosynthetic apparatus from oxidative damage as well as by enhancing the production of UV-absorbing anthocyanin pigments (Neff and Chory, 1998; Keller et al., 2011; Shaikhali et al., 2012; Alvarez-Fernandez et al., 2021).

CRYs have been implicated in other abiotic stress signaling pathways. CRY1 contributes to UV-B tolerance by regulating the re-dimerization of the UV-B receptor UV RESISTANCE LOCUS 8 (UVR8; Tissot and Ulm, 2020). In addition, stabilization of HY5 by CRYs *via* suppressing the COP1/SPA complex leads to the expression of genes that confer UV-B acclimation and tolerance. Indeed, both CRYs and UVR8 are essential for plant survival under UV-B, as the triple mutant lacking functional CRYs and UVR8 (*cry1 cry2 uvr8-2*) failed to survive under natural or simulated sunlight containing UV-B (Rai et al., 2019). CRYs were shown to enhance freezing tolerance when plants grown under blue light were acclimated to low, non-freezing temperatures before exposure to subzero temperatures (Li et al., 2021). CRYs are also important for drought tolerance, due to their functions in regulating stomatal aperture in concert with PHOTs and ABA (Mao et al., 2005; Boccalandro et al., 2012; Chen et al., 2012). Besides regulating stomatal aperture, CRYs promote the differentiation of stomata from protodermal cells which is promoted by light; the *cry1 cry2* mutant showed a lower stomatal density in blue light (Kang et al., 2009).

Besides abiotic stresses, CRYs have been implicated in biotic stress signaling pathways. CRYs enhance the resistance against bacterial and viral pathogens that attack the plants. CRY1 activates the systemic acquired resistance (SAR) locally in leaves infected with the bacteria *Pseudomonas syringae*, under continuous light conditions. In line with this, the expression levels of *PATHOGENESIS-RELATED GENE 1* (*PR1*) induced by salicylic acid treatment were enhanced in CRY1-overexpressing plants but decreased in *cry1* mutants (Wu and Yang, 2010). Similarly, Arabidopsis CRY2 and PHOT2 promote resistance (R) protein-mediated defense during Turnip Crinkle Virus (TCV) infection by stabilizing the R protein HYPERSENSITIVE RESPONSE TO TCV (HRT). In addition, HRT may function as a part of the protein complex consisting of CRYs and the E3 ligase CONSTITUTIVE PHOTOMORPHOGENIC 1 (COP1; Jeong et al., 2010a,b). CRY1 along with PHOT2 and PHYB was also shown to promote resistance against Cucumber Mosaic Virus (CMV) in Arabidopsis (Zhou et al., 2017).

After vegetative growth, Arabidopsis plants enter reproductive development through flowering, fertilization, and seed set. As an annual species not capable of clonal propagation, seed production is essential for the survival of this species. For successful reproduction, the timing of flowering is extremely crucial and needs to occur during a favorable season. Flowering time is therefore strongly regulated by environmental factors including day length. Long days, as in spring and summer, promote Arabidopsis flowering through a cryptochrome photoreceptor. The mechanisms allowing cryptochromes to sense the length of the day are well-described in (Wang and Lin, 2020a). The flowering of Arabidopsis is associated with the production of multiple, usually branched, inflorescences along which flowers are produced. The extent of shoot branching is highly controlled by many environmental factors including light. While phytochrome B promotes branching, CRY1 inhibits branching and, thus, acts oppositely to phytochrome (González-Grandío et al., 2013; Zhai et al., 2020).

## The Cryptochrome Photoreceptors CRY1 and CRY2: Structure and Functions

The Arabidopsis genome contains three *CRY* genes, two CRYs (CRY1 and CRY2) of the plant CRY subfamily, and one CRY (CRY3) of the CRY-DASH subfamily. CRY1 and CRY2 have proven signaling activities in photomorphogenesis, while CRY3 repairs cyclobutane pyrimidine dimers in UV-damaged single-stranded DNA and is localized in chloroplasts and mitochondria (Kleine et al., 2003; Selby and Sancar, 2006; Pokorny et al., 2008; Kiontke et al., 2020). Plant and animal-type CRYs evolved independently from different types of DNA photolyases; both have lost photolyase activity in the course of their evolution into photoreceptors (Sancar, 2003).

CRY1 and CRY2 have overlapping, but also distinct functions, as defined through the phenotypic analysis of *cry1* and *cry2* mutants. Both CRY1 and CRY2 have important functions during seedling deetiolation in blue light, with CRY1 acting mostly at higher fluence rates of blue light, while CRY2 responds primarily to dim blue light (Ahmad and Cashmore, 1993; Lin et al., 1998). This functional distinction correlates with a difference in photoreceptor stability: CRY2 is very photo-labile; it is degraded within a few hours of exposure to blue light. In contrast, CRY1 is mostly light-stable, though it was recently shown to be partially destabilized at very high fluence rates of blue light (Lin et al., 1998; Miao et al., 2021). Another major functional distinction is the regulation of flowering time: CRY2 is a key inducer of flowering under the long-day conditions that promote flowering in Arabidopsis, while the role of CRY1 is only of minor importance and dependent on the respective environmental conditions used (Guo et al., 1998; Mockler et al., 2003; Exner et al., 2010; Liu et al., 2016).

CRY1 and CRY2 consist of two domains, the highly conserved N-terminal photolyase homology region (PHR) and the more divergent, unstructured CRY Carboxy Terminus (CCT, also called CCE domain). The PHR domains of CRY1 and CRY2 (PHR1 and PHR2, respectively) non-covalently bind the primary chromophore, a flavin, and are therefore responsible for light detection by the photoreceptors (Wang and Lin, 2020a). Besides, PHRs mediate oligomerization of CRYs which is essential for CRY activities (Sang et al., 2005; Rosenfeldt et al., 2008; Wang et al., 2016a; Liu et al., 2020). The 3D structure of monomeric PHR1 was resolved several years ago, demonstrating high structural similarity with DNA photolyases (Brautigam et al., 2004). The crystal structure of PHR2 uncovered that its structure is almost identical to that of PHR1 (Ma et al., 2020b). Recently, three pivotal studies resolved the structure of the active PHR2 conformation either by X-ray crystallography or cryogenic electron microscopy (cryo-EM). All three studies independently demonstrated that the blue light-activated photosensory domain of CRY2 assembles into a tetramer (Shao et al., 2020; Ma et al., 2020a; Palayam et al., 2021).

The CCT domains of CRYs are intrinsically disordered; therefore, no structural information on CCT or full-length CRYs is so far available. The CCT domains of CRY1 and CRY2 share only weak sequence similarities and are of different lengths (Lin et al., 1998). CCTs are not directly involved in the perception of blue light but are important hubs for the blue light-induced interaction with signaling proteins. While the PHR domains were initially thought to solely act in photo-sensing, it has become clear that they also represent important docking domains for signal transduction *via* protein–protein interactions. Table 1 shows the list of proteins that interact with the PHR domain of CRYs and the respective signaling pathways involved.

**Table 1:** Proteins that interact with the N-terminal domains of Arabidopsis CRYs (PHR1 and PHR2).

Name	AGI code	Identity	Interaction with		Functions in	References
			PHR1	PHR2		
AGB1	AT4G34460	Heterotrimeric G protein beta subunit	Yes	Nd	Organ shape	Lian et al., 2018
ARF6	AT1G30330	Auxin response factor	Yes	Nd	Cell and organ elongation	Mao et al., 2020
ARF8	AT5G37020	Auxin response factor	Yes	Nd	Cell and organ elongation	Mao et al., 2020
ARP6	AT3G33520	Actin-related protein	Yes	Yes	Chromatin remodeling	Mao et al., 2021
BEE2	AT4G36540	Brassinosteroid signaling protein	Yes	Nd	Brassinosteroid signaling, shade avoidance	Wang et al., 2018a
BIC1	AT3G52740	Protein inhibiting CRY function	Yes	Yes	Disrupting CRY dimerization	Wang et al., 2016a; Ma et al., 2020b; Miao et al., 2021
BIC2	AT3G44450	Protein inhibiting CRY function	Yes	Yes	Disrupting CRY dimerization	Wang et al., 2016a; Ma et al., 2020b
BIM1	AT5G08130	bHLH protein	Yes	Yes	Brassinosteroid signaling	Wang et al., 2018b
BZR1	AT1G75080	Positive regulator of brassinosteroid signaling	Yes	Yes	Brassinosteroid signaling	He et al., 2019
BZR2 (BES1)	AT1G19350	Brassinosteroid signaling protein	Yes	Yes	Brassinosteroid signaling	Wang et al., 2018b; He et al., 2019
CIB1	AT4G34530	CRY-interacting bHLH protein	Yes	Nd	Flowering time	Liu et al., 2008; Wang et al., 2018a
CIL1	AT1G68920	CIB1-like bHLH protein	Yes	Nd	Photomorphogenesis	Wang et al., 2018a
CRY1	AT4G08920	Cryptochrome	Yes	Yes	Blue light perception	Sang et al., 2005; Liu et al., 2020
CRY2	AT1G04400	Cryptochrome	Yes	Yes	Blue light perception	Sang et al., 2005; Wang et al., 2016a
GAI	AT1G14920	DELLA protein	Yes	Nd	GA signaling	Zhong et al., 2021; Xu et al., 2021a
GID1	AT3G05120	DELLA protein	Yes	Nd	GA signaling	Zhong et al., 2021; Xu et al., 2021a
HBI1	AT2G18300	bHLH protein	Yes	Nd	Cell elongation and proliferation, plant immunity	Wang et al., 2018a
IAA12	AT3G23050	Auxin response protein	Yes	Nd	Auxin signaling	Xu et al., 2018
IAA17	AT1G04550	Auxin response protein	Yes	Nd	Auxin signaling	Xu et al., 2018
IAA7	AT1G04250	Auxin response protein	Yes	Nd	Auxin signaling	Xu et al., 2018
MTA	AT4G10760	mRNA m6A writer protein	Nd	Yes	N6-adenosine methylation of mRNA	Wang et al., 2021b
PIF5	AT3G59060	bHLH transcription factor	Nd	Yes	Shade avoidance, PHY signaling	Pedmale et al., 2016
RGA	AT2G01570	DELLA protein	Yes	Nd	GA signaling	Yan et al., 2021; Zhong et al., 2021; Xu et al., 2021a
SINAT2	AT3G58040	RING finger domain protein	Yes	Nd	Brassinosteroid signaling, photomorphogenesis	Hu et al., 2021
SINAT5	AT5G53360	RING finger domain protein	Yes	Nd	Brassinosteroid signaling, photomorphogenesis	Hu et al., 2021
SPA1	AT2G46340	Component of COP1/SPA ubiquitin ligase	No	Yes	Photomorphogenesis, flowering time	Lian et al., 2011; Liu et al., 2011; Zuo et al., 2011
SWC6	AT5G37055	Component of SWR1 complex	Yes	Yes	Chromatin remodeling, Flowering time	Mao et al., 2021
TCP17	AT5G08070	TCP family protein	Yes	Nd	Leaf differentiation	Zhou et al., 2019
TOE1	AT2G28550	AP2 family transcription factor	Yes	Yes	Flowering time, innate immunity	Du et al., 2020
TOE2	AT5G60120	AP2 family transcription	Yes	Yes	Flowering time, innate immunity	Du et al., 2020

*Nd, not determined*.

## Early Events in Cryptochrome Signal Transduction

**Figure 2:**
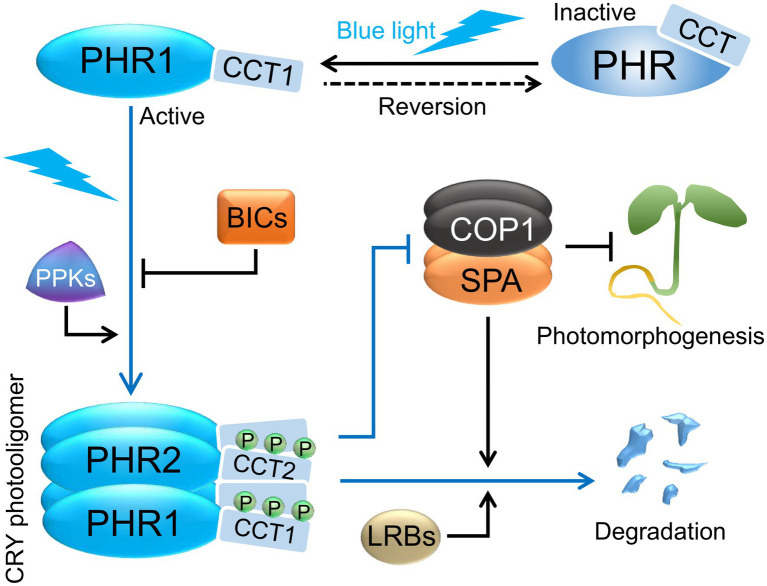
Cryptochrome photoactivation and degradation. Cryptochromes exist as inactive monomers in darkness and are proposed to form a “closed” conformation with the C-terminal domains (CCTs) closely associated with the N-terminal domains (PHRs). In blue light, cryptochromes homo- and heterooligomerize *via* their PHR domains, and serine residues in the CCTs are phosphorylated by PPK kinases. In addition, the CCTs are proposed to dissociate from PHRs forming an “open” conformation. Photoactivated CRYs revert to inactive forms in darkness in a temperature-dependent manner, which may help in retaining their photoresponsiveness. In blue light, BICs suppress CRY photooligomerization and thereby affect CRY function. Exposure to blue light ultimately leads to the degradation of CRY2, and a lesser extent of CRY1, *via* the COP1/SPA and LRB ubiquitin ligases. CRYs promote photomorphogenesis in blue light *via* repressing COP1/SPA activity. Abbreviations: PHR, photolyase homology region; CCT, CRY carboxy terminus; BIC, BLUE LIGHT INHIBITOR OF CRYPTOCHROMES; PPK, PHOTOREGULATORY PROTEIN KINASE; COP1, CONSTITUTIVE PHOTOMORPHOGENIC 1; SPA, SUPPRESSOR OF PHYA-105 1; LRB, Light-Response Bric-a-Brack/Tramtrack/Broad.

Upon light perception, CRYs undergo a conformational change that leads to oligomerization from a monomer to a tetramer (Ma et al., 2020a; Shao et al., 2020; Palayam et al., 2021; Figure 2). Oligomerization of CRY1 and CRY2 is light-dependent *in vivo*, with CRY2 being about six times more sensitive to blue light than CRY1 (Liu et al., 2020). This finding correlates with the phenotypic analyses of *cry1* and *cry2* mutants that identified CRY1 as a “higher blue light intensity” and CRY2 as a “lower blue light intensity” photoreceptor, suggesting that the different kinetics of photooligomerization are causative for the different blue light sensitivity of CRY1 and CRY2, in addition to the differential photoreceptor stability in blue light (Liu et al., 2020). Photooligomerization is indeed required for CRY1 and CRY2 activity *in vivo* (Sang et al., 2005; Rosenfeldt et al., 2008; Wang et al., 2016a). Recent results suggest that photooligomerization is necessary but not sufficient for CRY2 function *in vivo* because the expression of a CRY2 missense mutant (CRY2^P532L^) failed to complement the *cry1 cry2* mutant phenotype despite normal photooligomerization. Hence, the light response likely requires additional conformational or biochemical changes beyond photooligomerization (Liu et al., 2020). Initially, it was assumed that CRY1 and CRY2 can only homo-oligodimerize, but recently it was shown that CRY1 and CRY2 also form hetero-oligomers. Functional relevance of CRY1/CRY2 heterooligomer action was provided by showing that expression of a truncated CRY2 lacking the functionally important CCT domain (PHR2) in transgenic plants caused a dominant-negative effect by competitively inhibiting homodimerization of both endogenous CRY1 and CRY2. In accordance, overexpression of PHR2 repressed the activities of both endogenous CRY1 and CRY2 during deetiolation (Liu et al., 2020).

The lifetimes of cryptochrome signaling states are limited due to thermal dark reversion (Herbel et al., 2013). Such inactivation mechanisms are important to retain the photoresponsiveness of photoreceptors. Photooligomers of CRY2 revert to inactive monomers when transferred from blue light to darkness in a temperature-dependent fashion. Dark reversion of CRY2 was faster in Arabidopsis plants when compared to CRY2 heterologously expressed in mammalian HEK293 cells, suggesting that additional plant factors enhance CRY2 dark reversion (Liu et al., 2020; Wang and Lin, 2020a; Figure 2). Plant factors that reduce CRY1 and CRY2 oligomerization were identified in a genetic screen for negative regulators of CRY activity. Mutations in plant-specific *BLUE LIGHT INHIBITOR OF CRYPTOCHROMES 1* (*BIC1*) and the related gene *BIC2* enhance cryptochrome responses in Arabidopsis, while overexpression of *BIC1* or *BIC2* strongly suppresses CRY functions in transgenic plants (Wang et al., 2016a). BIC1 interacts with PHR2 in a strongly blue light-enhanced fashion in Arabidopsis plants. When recombinantly expressed in mammalian HEK293 cells, BIC1 severely suppresses CRY2 self-association. Moreover, BIC1 leads to an inhibition of blue light-dependent molecular activities of CRY2, such as photobody formation, phosphorylation, degradation, and binding of signaling components, suggesting that BIC1 has a primary function in inhibiting CRY2 photooligomerization (Figure 2) which subsequently leads to the suppression of molecular and physiological CRY2 photoresponses (Wang et al., 2016a). Recently, it was shown that BIC1 also interacts with PHR1 in a blue light-dependent manner to inhibit CRY1 self-association and CRY1 degradation under high blue light fluence rates (Miao et al., 2021). Gene expression of *BIC1* and *BIC2* is induced by blue, red, and UV-B light, thereby providing a negative feedback mechanism of CRYs and phytochromes on CRY1 and CRY2 signaling (Wang et al., 2017; Tissot and Ulm, 2020).

The co-crystal structure of PHR2 with the CRY2-interacting domain of BIC2 provided mechanistic evidence for BIC2’s inhibitory activity on CRY2 oligomerization (Ma et al., 2020b; Palayam et al., 2021). BIC2-binding did not change the overall conformation of the PHR2 tetramer, but caused structural rearrangements in side chains of amino acids that are considered important for photoreduction of the chromophore. Hence, BIC2 may prevent photoreduction of CRY2 which subsequently would inhibit photooligomerization in blue light (Ma et al., 2020b; Wang and Lin, 2020b). BIC2 might also act by a second mechanism involving competitive inhibition of CRY2 oligomerization. BIC2 occupies part of the oligomeric interaction surface of PHR2 and might thereby prevent photooligomerization of PHR2 (Ma et al., 2020b; Wang and Lin, 2020b; Palayam et al., 2021).

Interestingly, a recent study uncovered an additional function of BIC1 as a co-activator of brassinosteroid-induced gene expression and hypocotyl elongation in blue light. BIC1 interacts with the transcription factors BRASSINAZOLE-RESISTANT 1 (BZR1) and PHYTOCHROME-INTERACTING FACTOR 4 (PIF4) to cooperatively bind and activate BZR1 target genes involved in cell elongation. Hence, BIC1 may integrate cryptochrome and brassinosteroid signaling (Yang et al., 2021). The physical interaction of CRYs with BZR1 and other brassinosteroid signaling proteins provided further support for the direct regulation of brassinosteroid responses by blue light (Wang and Lin, 2020a). A recent finding that CRY2 activates gene expression by directly binding to DNA when expressed in a heterologous non-plant system even points toward a possible role of CRY2 as a transcription factor (Yang et al., 2018). Arabidopsis CRY2 reconstituted in human embryonic kidney cells (HEK293T) could directly interact with many fragments of the *FLOWERING LOCUS T* (*FT*) genomic region, in a blue light-enhanced manner. In contrast, CRY2 was associated only weakly with *FT* in transgenic plants, perhaps due to the presence of endogenous CRY-feedback mechanisms, such as the blue light-dependent degradation and BIC-mediated suppression (Yang et al., 2018). The idea of CRY2 binding DNA is conceivable, as CRYs have evolved from ancestral photolyases that bind pyrimidine dimers in the DNA. In line with this, the physical association of CRY2 with the genomic regions was also shown using ChIP-seq experiments in transgenic Arabidopsis lines (Pedmale et al., 2016). However, as CRY2 lacks the typical features of a canonical transcription factor, further experiments are needed to confirm the transcriptional activity of CRY2 in planta.

Following blue light absorption, CRY1 and CRY2 are phosphorylated (Shalitin et al., 2002, 2003; Yu et al., 2007a; Figure 2). There are three serines in the CCT domain of CRY2 and mutations in these serines reduce CRY2 phosphorylation, degradation, and seedling deetiolation in transgenic plants, indicating that light-induced phosphorylation is important for CRY2 activity in the context of a full-length CRY2 photoreceptor (Wang et al., 2015). Experiments expressing deletion-derivatives of CRY2 strongly suggest that light-induced phosphorylation of CRY2 is not important for CRY2 signaling activity *per se* but causes conformational changes that expose the CCT domain of CRY2 to render it accessible for important interacting signaling partners. Phosphorylation of CCT might provide a negative charge that repels CCT from the PHR domain (Yu et al., 2007b). Structural information of full-length CRY2 in its active and inactive conformation will be necessary to prove that blue light converts CRY2 from a “closed” into an “open” conformation (Figure 2). A recent work involving in-cell infrared difference (ICIRF) spectroscopy provides further evidence to the idea that blue light illumination causes the dissociation of CCT from PHR. Under darkness, in the full-length CRY from *Chlamydomonas reinhardtii* expressed in bacteria, the CCT was in close association with the only β-sheet of the PHR domain. Blue light illumination caused a re-organization of the PHR domain β-sheet due to the stabilization of flavin neural radical in presence of ATP, implying the dissociation of CCT from PHR (Goett-Zink and Kottke, 2021).

CRY2 is phosphorylated by a family of four related Ser/Thr protein kinases, the PHOTOREGULATORY PROTEIN KINASES (PPK1–PPK4; Figure 2). The major phosphorylation sites in CRY2 identified *via* mass spectrometry are located within the CCT domain of CRY2. *ppk* mutants exhibit impaired CRY2 phosphorylation and late-flowering similar to a *cry2* mutant, which supports that blue light-induced CRY2 phosphorylation is important for CRY2 activity (Liu et al., 2017). CRY2 also interacts with SUPPRESSOR OF PHYA-105 1 (SPA1) that was recently shown to have kinase activity (Paik et al., 2019). Whether SPA1 phosphorylates CRY1 or CRY2 is unknown. But CRY1 and CRY2 in *spa* null mutants exposed to blue light retain a normal, retarded migration in protein gels, suggesting that CRY phosphorylation still occurs in the absence of SPA proteins (Holtkotte et al., 2017).

After light-induced phosphorylation, CRY2 is rapidly degraded through polyubiquitination by the COP1/SPA and the Light-Response Bric-a-Brack/Tramtrack/Broad (LRB) E3 ubiquitin ligases. While the COP1/SPA complex particularly mediates the rapid response after 1–2 h of blue light exposure, LRBs are important for CRY2 degradation after more prolonged blue light irradiation (Shalitin et al., 2002; Weidler et al., 2012; Chen et al., 2021; Figure 2). Interestingly, CRY2 degradation is enhanced by low ambient temperature (16°C) through the polyubiquitination by LRBs (Ma et al., 2021). In contrast, CRY2 degradation is almost absent at 4°C when plants are acclimated to low temperatures to acquire freezing tolerance (Li et al., 2021). This may be caused by an enhanced shuttling of COP1 out of the nucleus and/or by a generally reduced activity of proteasomes at low temperatures (Catala et al., 2011; Li et al., 2021). Both COP1 and LRBs were also shown to control CRY1 protein stability under high blue light fluence rates (Miao et al., 2021).

## Signal Transduction Through Cryptochrome-Interacting Proteins

Cryptochrome signal transduction occurs *via* both the CCT and the PHR domains. Two decades ago, a key experiment had already provided strong evidence that CCT is sufficient to promote photomorphogenesis. Researchers fused the CCT domains of CRY1 and CRY2 to an artificial dimerization domain. Transgenic plants expressing these fusion proteins exhibited constitutive photomorphogenesis even in darkness, indicating that CCT in the absence of the photosensory PHR constitutively activates the light signaling cascade (Yang et al., 2000). While PHR was initially thought to act exclusively in photoperception, there is mounting evidence that PHR also initiates important signaling processes because expression of CRY1-PHR fused to an artificial nuclear localization sequence complemented the *cry1* mutant phenotype (He et al., 2015). Moreover, many signaling factors interact with PHRs (see also below; Table 1). How PHR and CCT coordinate light signaling is so far unknown, but a comparative transcriptome analysis of PHR and CCT-expressing transgenic lines provided insight into the co-regulation of genes by both domains. While approximately 67% of CRY1-responsive genes are regulated by CCT1 alone, others are co-regulated by both CCT1 and PHR1 (Wang et al., 2016b).

### The COP1/SPA Ubiquitin Ligase

Loss-of-function missense mutations in the CCT domains of CRY1 and CRY2 confirmed CCTs as signaling hubs (Ahmad and Cashmore, 1993; Ahmad et al., 1995; Guo et al., 1998; Yang et al., 2000). The CCT domains of CRY1 and CRY2 interact with COP1 (Wang et al., 2001; Yang et al., 2001; Ponnu et al., 2019), an E3 ubiquitin ligase that inhibits light signaling by polyubiquitinating multiple transcription factors acting in the various light responses. COP1 is mainly active in darkness, thereby preventing an accumulation of these substrate transcription factors which leads to a suppression of light responses in darkness (Huang et al., 2014; Hoecker, 2017; Ponnu and Hoecker, 2021). Epistatic analyses of *cop1* and *cry1* mutants suggested early on that CRYs initiate light signaling in blue light by inhibiting the activity of COP1 (Ang and Deng, 1994; Figures 1, 2). Indeed, substrate transcription factors of COP1 accumulate in seedlings grown in blue light, thereby allowing photomorphogenesis to proceed (Ponnu, 2020).

In darkness, COP1 acts in concert with SPA proteins in a tetrameric COP1/SPA complex consisting of two COP1 proteins and two SPA proteins of the four-member SPA protein family (SPA1-SPA4; Hoecker and Quail, 2001; Saijo et al., 2003; Zhu et al., 2008). Both COP1 and SPA proteins are necessary for the activity of the COP1/SPA ubiquitin ligase since both *cop1* and *spa* quadruple mutants undergo constitutive photomorphogenesis, exhibiting features of light-grown seedlings in complete darkness (Deng et al., 1991; Laubinger et al., 2004; Ordonez-Herrera et al., 2015). The COP1/SPA complex acts as the substrate recognition module in a Cullin 4-based ubiquitin ligase. Substrate recognition occurs *via* the C-terminal WD-repeat domains present in both the COP1 and the four SPA proteins. The WD repeats also act in binding DDB1 in the Cullin 4-complex (Ponnu and Hoecker, 2021). Since the WD-repeat of SPA1 can replace that of COP1 but not vice versa, their functions are likely related but not identical (Kerner et al., 2021). COP1/SPA complex formation is conferred by the coiled-coil domains present in COP1 and SPA proteins. The N-termini of COP1 and SPA proteins are distinct, with COP1 carrying a RING finger domain and SPA exhibiting a kinase-like domain that was recently shown to have kinase activity (Deng et al., 1992; Hoecker et al., 1999; Paik et al., 2019; Ponnu and Hoecker, 2021; Wang et al., 2021a).

CRY1 and CRY2 interact with the COP1/SPA complex in blue light-grown but not in dark-grown seedlings *in vivo* (Holtkotte et al., 2017), which is consistent with the blue light-induced inhibition of COP1/SPA activity by CRYs. Moreover, the COP1/SPA complex preferentially interacts with the phosphorylated isoforms of CRY1 and CRY2 that only exist after blue light exposure (Holtkotte et al., 2017). When examining one-by-one interactions *ex vivo*, CRY1 and CRY2 interact with SPA1 in a blue light-dependent manner, while they interact with COP1 constitutively or in a blue light-enhanced manner (Yang et al., 2001; Lian et al., 2011; Liu et al., 2011; Zuo et al., 2011; Ponnu et al., 2019). This suggests that SPA1 may contribute to the blue light dependency of the CRY-COP1/SPA interaction *in vivo*. Indeed, SPA proteins are required for the CRY1-COP1/SPA interaction *in vivo* because COP1 did not interact with CRY1 in a *spa* quadruple null mutant (Holtkotte et al., 2017).

Domain mapping defined the interacting domains in CRYs and the COP1 and SPA proteins, thereby helping in the elucidation of the molecular mechanisms of CRY activity. The CCT domains of CRY1 and CRY2 interact with the substrate-recognizing WD repeats of COP1 (Wang et al., 2001; Yang et al., 2001; Ponnu et al., 2019). Binding of CRY1 and CRY2 to SPA proteins differs: The CCT domain of CRY1 interacts with SPA1WD (Lian et al., 2011; Liu et al., 2011; Ponnu et al., 2019), while the CCT domain of CRY2 does not interact with full-length SPA1 but with an N-terminally truncated version of SPA1 containing only the WD-repeat domain, suggesting that the N-terminal domain of SPA1 inhibits the CCT2-SPA1WD interaction (Ponnu et al., 2019). CRY2 interacts most strongly *via* its PHR domain with the kinase domain of SPA1 (Zuo et al., 2011).

CRYs inactivate the COP1/SPA complex *via* multiple mechanisms (Ponnu, 2020). Light promotes the nuclear exclusion of COP1, thereby separating it from the nuclear-localized transcription factors targets (Von Arnim and Deng, 1994; Pacin et al., 2014). In blue light, nuclear exclusion of COP1 is dependent on CRY1 (Osterlund and Deng, 1998). Nuclear exclusion of COP1 in white light, but not in blue light, is dependent on SPA proteins (Balcerowicz et al., 2017). Blue light leads to a destabilization of SPA1 and SPA2, but this occurs in a phytochrome A-dependent fashion and is independent of CRYs (Balcerowicz et al., 2011; Chen et al., 2015). Light-activated CRY1 acts to disrupt the COP1-SPA1 interaction (Lian et al., 2011; Liu et al., 2011). CRY2 does not dissociate the COP1-SPA1 complex, but blue light enhances the CRY2-COP1 interaction, which may lead to a stronger inactivation of the COP1/SPA complex (Zuo et al., 2011).

Two recent, complementary studies demonstrated that photoactivated CRY2 inactivates the COP1/SPA complex by competitively displacing substrates from the WD-repeat domain of COP1 (Lau et al., 2019; Ponnu et al., 2019; Ponnu, 2020). This mechanism is based on the sharing of a COP1-interacting motif between CRY2 and COP1 substrates. COP1 recognizes many diverse substrate transcription factors; despite their sequence divergence, many of them share a conserved Val-Pro (VP) motif that is required for their interaction with COP1 (Holm et al., 2001; Ponnu et al., 2019). The co-crystal structure of COP1 with VP-containing peptides confirmed that the VP directly binds the WD-repeat domain of COP1 (Uljon et al., 2016; Lau et al., 2019). CRY2 also contains a VP-like motif in its CCT domain, suggesting that CRY2 and substrates may compete for binding to the same domain of COP1 (Muller and Bouly, 2015). Indeed, when the VP motif of CRY2 was mutated, the interaction with COP1 was lost (Ponnu et al., 2019). In agreement with this finding, CRY2 carrying a mutated VP motif failed to complement the *cry2* mutant phenotype and was, therefore, inactive in Arabidopsis plants (Ponnu et al., 2019; Liu et al., 2020). In addition, loss of function of CRY2 was observed when the proline residue of the VP motif was mutated (CRY2^P532L^; Ahmad et al., 1995; Liu et al., 2020). A co-crystal structure of COP1WD with a VP-containing peptide of CRY2 confirmed that the VP of CRY2 binds COP1 (Lau et al., 2019). Hence, both COP1 substrates and CRY2 bind COP1WD *via* their respective VP motifs. Competitive binding was investigated using the COP1-substrate PRODUCTION OF ANTHOCYANIN PIGMENT 2 (PAP2), a transcription factor that controls the biosynthesis of anthocyanins in light-grown plants (Maier et al., 2013; Maier and Hoecker, 2014). Co-expression of CRY2 disrupted the COP1-PAP2 interaction, indicating that CRY2 effectively displaced PAP2 from COP1. In contrast, CRY2 carrying the VP mutation was not able to disrupt the COP1-PAP2 interaction (Ponnu et al., 2019). Co-crystals of COP1WD with a VP-peptide from the COP1-substrate CONSTANS (CO) and the corresponding late-flowering phenotype of plants expressing COP1 carrying WD mutations disrupting VP-binding suggest that CRY2 may also outcompete CO from binding to COP1 (Lau et al., 2019). Recently, co-expression of CRY2 was also shown to reduce the COP1-ELONGATED HYPOCOTYL 5 (HY5) interaction in a split-luciferase assay (Li et al., 2021).

Though the CCT motifs of CRY1 and CRY2 share little sequence similarity, CRY1 does contain three potential VP motifs in its CCT domain of which the first one (VP1) is required for COP1-binding (Ponnu et al., 2019). A peptide containing VP1 of CRY1 was also co-crystallized with COP1WD, thus confirming a direct interaction of VP1 with COP1 (Lau et al., 2019). It remains to be determined whether CRY1, like CRY2, inactivates COP1 by substrate displacement. Moreover, it is unknown whether CRYs displace substrates also from the WD repeats of SPA proteins. Considering that both the light-activated CRYs and the COP1/SPA complex act as tetramers, it is conceivable that a cryptochrome tetramer displaces substrates from the COP1/SPA hetero-tetramer.

### Cryptochrome-Interacting Transcriptional Regulators

**Figure 3:**
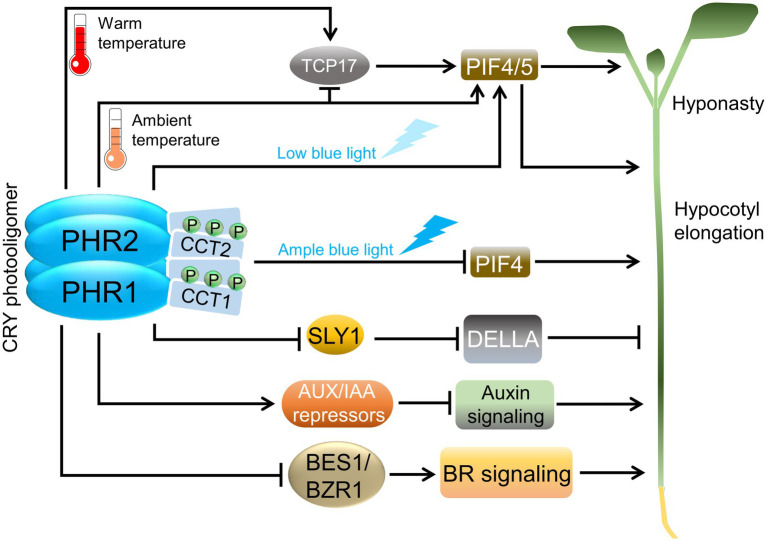
Cryptochromes in warm temperature and hormone signaling. Abbreviations: PHR, photolyase homology region; CCT, CRY carboxy terminus; TCP17, TCP DOMAIN PROTEIN 17; SLY1, SLEEPY1; AUX/IAA, AUXIN/INDOLE-3-ACETIC ACID; BES1, BRI1-EMS-SUPPRESSOR 1; BZR1, BRASSINAZOLE-RESISTANT 1; PIF, PHYTOCHROME-INTERACTING FACTOR; DELLA, DELLA domain-containing protein; BR, Brassinosteroid.

Besides regulating transcription factor activity indirectly *via* the COP1/SPA ubiquitin ligase, CRYs also directly interact with several transcription factors to initiate light signal transduction (Wang and Lin, 2020a). PHR2 of photoactivated CRY2 interacts with a family of CRYPTOCHROME-INTERACTING bHLH (CIB) transcription factors to regulate flowering time in Arabidopsis. Similarly, PHR1 interacts with CIB proteins to regulate seedling deetiolation (Wang and Lin, 2020a). This finding supports the observation that overexpression of PHR1 can initiate light signaling in the absence of CCT (He et al., 2015). CRY1 and the PHR domain of CRY2 also interact with the PHYTOCHROME-INTERACTING FACTORS PIF4 and PIF5 to control hypocotyl elongation under low blue light as it is found under a plant canopy (Pedmale et al., 2016; Xu et al., 2016; Figure 3). PIF4 and PIF5 belong to a small bHLH transcription factor family that promotes the expression of cell elongation-related genes in darkness and shade (Balcerowicz, 2020). In the regulation of flowering time, photoactivated CRY2 directly interacts with the floral repressors TARGET OF EARLY ACTIVATION TAGGED 1 (TOE1) and TOE2 to suppress their repressor activities on the floral activator CO (Du et al., 2020).

In seedlings, the hypocotyls do not only elongate in response to darkness or under a canopy but also when exposed to a moderate increase in ambient temperature that is not yet heat stress to the plant (*ca.* 28°C). Blue light inhibits the elongation response to high ambient temperature through an interaction of CRY1 with PIF4, thereby causing a reduction in the transcription activation function of PIF4 (Ma et al., 2016; Xu et al., 2016). CRY1 also controls PIF4 activity in a temperature-dependent manner through the transcription factor TCP DOMAIN PROTEIN 17 (TCP17): PHR1 interacts directly with TCP17 at ambient temperature, thereby inhibiting TCP17 activity (Figure 3). At high ambient temperature, the CRY1-TCP17 interaction is reduced and TCP17 can activate *PIF4* gene expression and—after direct binding to the PIF4 protein—the TCP17/PIF4 complex activates cell elongation-related genes, leading to hypocotyl elongation (Zhou et al., 2019).

CRYs mediate blue light control of hormone responses by directly interacting with several hormone signal transduction intermediates (Figure 3). Hormones are key regulators in plants as they—similar to light—control growth and development throughout the plant life cycle. Two very recent reports showed that CRY1 directly interacts with the receptor for the hormone gibberellic acid (GA; Zhong et al., 2021; Xu et al., 2021a). GA is known to be important for light responses; it induces cell elongation, and GA biosynthesis is required for the suppression of photomorphogenesis in darkness (Alabadí et al., 2004). In light-grown seedlings, GA biosynthesis and/or the action of GA signaling components are altered (Halliday and Fankhauser, 2003). Both recent studies demonstrate that blue light exposure induces the interaction of CRY1 with the GA receptor GID1 as well as with a family of GA signaling proteins, the DELLAs. DELLAs are repressors of GA signaling whose degradation is induced by GA through the action of the SLEEPY1, (SLY1)/SNE E3 ubiquitin ligases. Such de-repression subsequently allows GA-responsive gene expression and cell elongation (Xu et al., 2014). To analyze the consequences of CRY1-GID1-DELLA interactions, the scientists investigated DELLA degradation and found that blue light and CRY1 reduce the degradation of DELLAs in response to GA. It is expected that the increased DELLA levels in blue light contribute to the short hypocotyl phenotype of blue light-exposed seedlings. Consistent with this observation, GID1, DELLAs, and CRY1 genetically interacted in the control of hypocotyl elongation in blue light and GA responsiveness. To elucidate how CRY1 stabilizes DELLAs in blue light, protein–protein interactions were investigated. Two mechanisms were identified: CRY1 inhibits the GID1-DELLA interaction, thereby preventing GA-induced DELLA degradation (Xu et al., 2021a; Zhong et al., 2021). Furthermore, CRY1 disrupts the interaction of DELLAs with the SLY1 E3 ubiquitin ligase that targets DELLAs (Xu et al., 2021a). Taken together, these results show that blue light perceived by CRY1 antagonizes GA-induced cell elongation by enhancing the levels of DELLA repressors. DELLA stability is also controlled by a second E3 ubiquitin ligase (COP1/SPA) in a shade- and temperature-dependent, but GA-independent manner (Blanco-Touriñán et al., 2020). Hence, DELLAs are likely stabilized in light-grown seedlings *via* COP1-dependent and COP1-independent mechanisms.

Auxin is a key hormone in photomorphogenesis and, in particular, in shade avoidance responses. Generally, it promotes cell elongation in darkness and canopy shade (Ma and Li, 2019). CRY1 represses auxin-induced cell elongation in blue light, thereby contributing to the inhibition of hypocotyl elongation in blue light. Treatment of seedlings with external auxin solutions indicated that photoactivated CRY1 inhibits the responsiveness of seedlings to auxin, suggesting that CRY1 inhibits auxin signal transduction (Xu et al., 2018). The nuclear auxin-signaling pathway is quite well-understood (Powers and Strader, 2020): auxin is perceived by a co-receptor complex consisting of an F-box protein of the TRANSPORT INHIBITOR RESPONSE 1/AUXIN-SIGNALING F-BOX (TIR1/AFB) family and a member of the AUXIN/INDOLE-3-ACETIC ACID (AUX/IAA) protein family. Upon binding of auxin, this co-receptor complex assembles, followed by polyubiquitination and degradation of the AUX/IAA repressor protein by the TIR1/AFB ubiquitin ligase. The degradation of AUX/IAA repressors activates auxin-responsive transcription factors (ARFs) to initiate gene regulation and the inhibition of cell elongation in response to auxin. CRY1 signaling directly interferes with the auxin-signaling pathway *via* at least two molecular mechanisms. It was found that the PHR1 directly binds AUX/IAA proteins in a blue light-dependent fashion, thereby inhibiting the interaction of the TIR1/AFB F-Box proteins with AUX/IAAs which causes stabilization of AUX/IAA proteins in the presence of auxin. Hence, blue light-activated CRY1 increases AUX/IAA repressor activity, thereby inhibiting cell elongation in blue light (Xu et al., 2018). In a second mechanism, CRY1 was found to interact with the transcription factors ARF6 and ARF8 in blue light. This interaction is also conferred by the PHR1 domain. The binding of PHR1 to ARF6 and ARF8 reduces the DNA-binding activity of these transcription factors *in vitro*. Moreover, *in vivo* chromatin immunoprecipitation experiments confirmed that blue light inhibits the binding of ARF6 and ARF8 to known target genes involved in cell elongation (Mao et al., 2020). In summary, these results indicate that CRY1 inhibits auxin signaling by stabilizing AUX/IAA repressors and by inhibiting the activity of ARF6/ARF8 activators of cell elongation genes. Interestingly, AUX/IAAs and ARF6/ARF8 were also found to interact with phytochrome B in red light, suggesting that phytochrome B and CRY1 share these mechanisms in promoting deetiolation in the light (Colón-Carmona et al., 2000; Xu et al., 2018; Mao et al., 2020).

CRYs also alter the response to the hormone class of brassinosteroids (BRs). BRs promote cell elongation and are required for seedling etiolation in darkness and shade (Lv and Li, 2020). For example, a seedling defective in BR biosynthesis or perception undergoes constitutive photomorphogenesis in darkness (Li et al., 1996; Li and Chory, 1997). CRYs inhibit BR signaling by direct interaction with BR signaling intermediates. CRY1 and CRY2 interact with the transcriptional activators BRI1-EMS-SUPPRESSOR 1 (BES1) and BZR1 to inhibit their activity in the BR-activation of gene expression. Moreover, CRY1 interacts with the kinase BRASSINOSTEROID-INSENSITIVE 2 (BIN2) which promotes the phosphorylation of BZR1 by BIN2, resulting in reduced BZR1 protein levels in the nucleus (Wang et al., 2018b; He et al., 2019). Hence, CRYs inhibit the levels and activities of two master regulators of BR-response, BES1, and BZR1.

### Cryptochrome-Interacting Chromatin Remodeling Proteins

A recent report provided evidence for a direct regulation of chromatin remodeling by CRY1 (Mao et al., 2021). Chromatin-remodeling or multi-dimensional alterations in chromatin architecture regulate gene expression by modifying the access of genomic DNA to the transcriptional regulatory machinery. Histone H2A.Z, a variant of histone H2A, modulates diverse cellular processes largely by regulating nucleosome composition and affecting DNA methylation. In eukaryotes, an exchange of H2A for H2A.Z by the SWI2/SNF2-RELATED (SWR1) chromatin-remodeling complex can lead to activation or repression of gene expression at the respective loci (Jarillo and Piñeiro, 2015; Aslam et al., 2019). CRY1 was shown to interact with two essential components of the SWR1 complex (SWC6 and ARP6) in a blue light-dependent manner, suggesting that chromatin remodeling may be important for photomorphogenesis. Indeed, *swc6*, *arp6*, and *h2a*.z mutant seedlings are hyposensitive to blue light, exhibiting longer hypocotyls than the wild type in blue light but not in darkness (Mao et al., 2021). Hence, H2A.Z histone deposition promotes photomorphogenesis by inhibiting the hypocotyl elongation in blue light. It is therefore hypothesized that CRY1 activates photomorphogenesis in part by promoting H2A.Z deposition. Mechanistically, this may occur by promoting SWR1 complex assembly because CRY1 enhances the interaction between SWC6 and ARP6. To address how SWR1 may be recruited to genes relevant for photomorphogenesis, it was tested whether SWR1 interacts with HY5, a key transcription factor in photomorphogenesis. It was found that SWC6, as well as ARP6, interact with HY5; moreover, CRYs, HY5, and SWC6/ARP6 promote H2A.Z deposition in blue light at selected HY5 target genes that are repressed by HY5 in blue light (Mao et al., 2021). Though HY5 mainly acts in transcription activation (Burko et al., 2020), it can also directly act in gene repression of these cell elongation-related genes (*IAA19*, *expansin*, *XTH*). Hence, the SWR1 complex may be critical in turning HY5 into a repressor of gene expression which subsequently contributes to the inhibition of hypocotyl elongation in blue light (Mao et al., 2021). Interestingly, chromatin-remodeling mutants are also hyposensitive to red and far-red light, indicating that the SWR1 complex is also important for phytochrome signaling. Further studies have shown that phytochrome B interacts with SWC6 and ARP6 (Mao et al., 2021; Wei et al., 2021).

### Cryptochromes, RNA Modification by N^6^ Methyladenosine, and Liquid–Liquid Phase Separation

The modification of RNAs by methylation of N^6^ adenosine (m^6^A) has emerged as an important regulatory mechanism. It is quite well-described in animals, while its effects are only beginning to be discovered in plants. In animals, m^6^A-methylation can affect RNA metabolism at several levels, including splicing, transcript stability, polyadenylation, RNA export, and translation (Reichel et al., 2019). m^6^A is deposited on RNA by so-called “writers,” a multiprotein complex with methylase activity. “Readers” interpret the code by binding to the m^6^A modification followed by posttranscriptional regulation, and “erasers” remove the m^6^A modification. Epitranscriptome analyses in mammals indicated that on average one m^6^A modification occurs every 2000 bp and, therefore, is quite frequent (Reichel et al., 2019).

Purification of CRY2-associated proteins by immunoprecipitation and mass spectrometry identified several m^6^A writer proteins. These interactions were confirmed by direct protein–protein interaction assays (Wang et al., 2021b). CRY2 interacted with the writers mRNA adenosine methylase (MTA), METHYLTRANSFERASE B (MTB), and FK506-BINDING PROTEIN 12 (FKBP12) INTERACTING PROTEIN 37 (FIP37) in heterologous HEK293 cells and in planta in split-YFP assays and co-immunoprecipitations. Domain mapping indicated that both PHR2 and CCT2 interacted with the writers. Interestingly, CRY1 also interacted with writers, though this interaction was not explored further. To assess the biological relevance of these interactions, the m^6^A epitranscriptome between wild-type and *cry1 cry2* mutant seedlings was compared in darkness and light. CRY-dependent m^6^A marks were found in particular in the 3′UTR of transcripts which is consistent with a general enrichment of m^6^A deposits in 3′UTRs in mammals (Reichel et al., 2019). In particular, RNAs of genes controlled by the circadian clock, such as *CCA1*, were enriched in blue light- and CRY-dependent m^6^A modification. The authors, therefore, hypothesized that m^6^A marks on clock-controlled transcripts may affect mRNA abundance and the circadian clock. Indeed, writer-defective *mta* mutants and *cry1 cry2* mutants exhibited reduced *CCA1* transcript stability in blue light, but not in darkness, when compared to the wild type, suggesting that m^6^A deposition enhances *CCA1* transcript stability in blue light. Consistent with this finding, *mta* mutants exhibited a longer period length under free-running, continuous white light conditions, similar to the *cry1 cry2* mutant. This phenotype, however, was lost when *mta* mutants were transferred to free-running blue light conditions which may be due to antagonistic functions of phytochromes and CRYs (Wang et al., 2021b).

While there are CRY-dependent m^6^A depositions on RNAs in blue light, the interaction of CRY2 with writer proteins was not blue light-dependent but also observed in darkness (Wang et al., 2021b). This raised the question of how blue light may control m^6^A modification through CRYs. Interestingly, it was found that the recruitment of the MTA protein into CRY2 photobodies was blue light-dependent, raising the possibility that MTA concentrations locally increase in CRY2 photobodies under blue light without changing the affinity for interacting proteins (Wang et al., 2021b). Photobodies have been described as membrane-less compartments that concentrate molecules through liquid–liquid phase separation (LLPS; Pardi and Nusinow, 2021). During LLPS, two liquid phases are formed, a denser and a less dense one, thereby locally changing the concentration of biomolecules without the need for membranes. LLPS condensates have certain characteristics (Emenecker et al., 2021; Xu et al., 2021b) that were found also in CRY2 photobodies, such as spherical shape, dynamic nature as shown by rapid photobody recovery after photobleaching, reversibility in darkness, and the capacity of smaller photobodies rapidly fusing to form larger ones. Taken together, these results suggest that CRY2 undergoes LLPS in blue light which leads to a local increase in the concentration of recruited MTA proteins. This may enhance m^6^A modification of RNAs in blue light (Quail, 2021; Wang et al., 2021b). Interestingly, the RNA-binding, flowering time regulator FLOWERING CONTROL LOCUS A (FCA) that contains prion-like domains shows LLPS behavior *in vivo* (Fang et al., 2019). Another prion-like domain-containing protein EARLY FLOWERING 3 (ELF3) was recently shown to undergo LLPS in response to high ambient temperatures (Jung et al., 2020). These pieces of evidence indicate that LLPS in plants is not limited to photobodies.

## Conclusion and Perspectives

Figures 1, 2 provide overviews of CRY photoactivation and signaling pathways along with the major downstream developmental responses. In addition to the CCTs, the PHR domains interact with many proteins that are integral to CRY functions (Table 1). While many fundamental questions starting from the exact mechanisms of CRY photoactivation and dark/thermal reversion remain to be answered, breakthroughs in recent years have immensely expanded our knowledge and presented us with more challenges.

Recent research showed that the active conformation of CRYs involve heterooligomers that are formed upon exposure to blue light. Besides CRY heterooligomers, the functional CRY supercomplexes may include many CRY-interacting proteins. An interesting question related to the dynamics of CRY complexes is: What are the mechanisms that regulate the formation and dissociation of such assemblies? As light responses are dissimilar at tissue and cell levels, the dynamics of the CRY complexes may be controlled at spatial levels. Temporal control of CRY supercomplexes can also be expected as tissues and organs at different maturity levels may respond differently to light signals. Changing light intensities under natural conditions may pose another layer of complexity. In particular, it is not well understood whether cryptochrome signaling enhances the fitness of plants under natural conditions. The LLPS nature of CRY photobodies may hold further clues. As CRY photobodies have been shown to act as hubs for mRNA methylation and gene expression, the influence of CRYs may not be restricted to the circadian genes and may regulate the transcriptional landscape in general. Advanced technologies, such as single-cell epigenomic and epitranscriptomic assays, may bring further breakthroughs in CRY research.

## Author Contributions

JP and UH wrote the manuscript. JP designed the figure and assembled Table 1. All authors contributed to the article and approved the submitted version.

## Funding

Our work on Arabidopsis light signal transduction is supported by the Deutsche Forschungsgemeinschaft (DFG) HO2793/3-4 and under Germany’s Excellence Strategy EXC2048/1 project ID 390686111 to UH.

## Conflict of Interest

The authors declare that the research was conducted in the absence of any commercial or financial relationships that could be construed as a potential conflict of interest.

## Publisher’s Note

All claims expressed in this article are solely those of the authors and do not necessarily represent those of their affiliated organizations, or those of the publisher, the editors and the reviewers. Any product that may be evaluated in this article, or claim that may be made by its manufacturer, is not guaranteed or endorsed by the publisher.
